# Beyond Screening: Can the Mini-Mental State Examination be Used as an Exclusion Tool in a Memory Clinic?

**DOI:** 10.3390/diagnostics5040475

**Published:** 2015-11-04

**Authors:** Xin Xu, Eddie Chong, Saima Hilal, Mohammad Kamran Ikram, Narayanaswamy Venketasubramanian, Christopher Chen

**Affiliations:** Memory, Aging and Cognition Center, Department of Pharmacology, National University Health System, Singapore 117600, Singapore; E-Mails: phcxx@nus.edu.sg (X.X.); eddie_chong@nuhs.edu.sg (E.C.); phchs@nus.edu.sg (S.H.); kamran.ikram@duke-nus.edu.sg (M.K.I.); drnvramani@gmail.com (N.V.)

**Keywords:** dementia, cognitive assessment, Mini-Mental State Examination, memory clinic

## Abstract

This study explores whether the Mini-Mental State Examination (MMSE) could reliably exclude definite dementia and dementia-free cases from requiring more extensive neuropsychological investigations in memory clinic settings in Singapore. Patients with memory complaints referred for possible dementia underwent the MMSE, followed by standardized neuropsychological and clinical assessments which led to a consensus diagnosis. MMSE cut-off points were derived stratified for education (less and equal/above primary level). Results show that after education stratification, using an optimal Positive Likelihood Ratio (PLR) and optimal Negative Likelihood Ratio (NLR), a higher percentage of patients were correctly identified as having dementia or dementia-free, with minimal misclassification rate. The finding suggests the MMSE can be used to exclude patients not requiring full neuropsychological assessments in a memory clinic.

## 1. Introduction

The Mini-Mental State Examination (MMSE) is a commonly administered screening tool for cognitive impairment and possible dementia in clinical settings with a high base rate of dementia [1,2,3]. The utility of the MMSE in detecting dementia and to assess disease progression has been shown to be valid and reliable [4,5]. Most previous studies investigated the utility of the MMSE as a dementia screening tool, where a comprehensive neuropsychological assessment was used to ascertain diagnosis. Hence, high sensitivity is the key in these studies in order to reduce the false negative rate. However this criterion has limited practical use in clinical settings where an efficient, effective and easily administered tool is needed to identify those who are most likely to be cognitively normal or dementia-free cases, hence avoiding the need to undergo a full neuropsychological assessment which utilizes scarce resources and also increases medical costs.

The MMSE has been criticized, in that it may be biased towards demographic characteristics such as age, educational level and ethnicity [6,7,8,9]. Advanced age and lower education were reported to exert significant negative impact on MMSE performance as well as on the discriminative value of the test itself [10,11,12,13]. Age and educational level specific cut-off scores have resulted in improved sensitivity compared to the traditional cut-off of 23 with limited loss of specificity [13].

In addition, a population based study in Singapore reported a higher positive likelihood ratio among participants with more than primary level education compared to participants with just a primary level of education or less (6.77 *vs.* 2.66) with a MMSE cut-off score of 23/24 in discriminating patients with or without dementia [14]. However, this adjustment for age and educational level requires further validation [15,16,17].

The present study aims to investigate the utility of the MMSE at a memory clinic setting in identifying patients who do not require further formal neuropsychological assessment for the diagnosis of dementia. Furthermore, we examined an education stratification strategy as suggested by two community based studies previously performed in Singapore [14,18].

## 2. Methods

### 2.1. Participants

A total of 724 consecutive patients attending the National University Hospital (NUH) memory clinic, Singapore, were assessed between 1 February 2006 and 30 April 2013. This study was approved by the Domain-Specific Review Board (DSRB) of the National Healthcare Group. A total of 716 patients were included in the analyses as three patients did not complete the MMSE (due to visual impairment) and five patients with moderate or severe depression were excluded.

### 2.2. Neuropsychological and Clinical Evaluation

We used a locally validated version of the MMSE which was previously used in Singapore-based studies [14,18]. The MMSE was administered independent of a comprehensive neuropsychological evaluation using the Repeatable Battery for the Assessment of Neuropsychological Status (RBANS) [19], 30-Item modified Boston Naming Test (mBNT) [20], and Color Trails Test (CTT) [21], performed by trained research psychologists. This formal neuropsychological battery measured cognitive performance on various domains such as immediate and delayed memory, visuoconstruction, language, attention and executive function.

An extensive uniform clinical assessment was conducted including physical examination, history taking, laboratory blood tests and computed tomography (CT) and/or magnetic resonance imaging (MRI) scans of the brain.

### 2.3. Diagnosis of Dementia

Diagnosis of dementia was made during weekly consensus meeting among three neurologists, two clinicians and five psychologists. Dementia and Mild Cognitive Impairment (MCI) were diagnosed according to standard clinical diagnostic criteria using the Diagnostic and Statistical Manual of Mental Disorders, Fourth Edition (DSM-IV) [22] and the revised Petersen’s diagnostic algorithm [23] respectively.

### 2.4. Determination on Optimal Cut-off Scores of the MMSE

As positive/negative predictive values (PPV/NPV) depend on the disease prevalence of the tested population, in a memory clinic sample, PPV is likely to be high simply due to high proportion of patients with dementia. Hence, likelihood ratio was utilized to identify the optimal MMSE cut-off scores as recommended in previous studies [24,25]. Likelihood ratio is the probability of a positive test outcome being found in persons with positive condition divided by the probability of positive test outcome being found in person with negative disease condition. The formula of calculating likelihood ratio is

Positive Likelihood Ratio (PLR) = Sensitivity/(1-Specificity)(1)

Negative Likelihood Ratio (NLR) = (1- Sensitivity)/Specificity(2)

A PLR of >5 indicates a large post-test probability of the existing disease state, in this case, dementia. Similarly, a NLR of <0.2 indicates a large post-test probability of the absence of dementia [25]. We adopted these indexes in the present study and chose MMSE cut-off scores with optimal PLR/NLR.

### 2.5. Statistical Analysis

Data analysis was carried out using SPSS 20.0 (IBM Corporation, Armonk, NY, USA). Chi-square tests were conducted to compare the differences, if any, of gender, ethnicity and educational level and independent *t*-test was used to compare the means of age, years of education, MMSE total score, between two groups: dementia and dementia-free.

ANOVA was conducted with *post hoc* analysis (Bonferroni test) to investigate the impact of demographic factors on MMSE performance. Receiver operating characteristic (ROC) analysis was used to determine MMSE-appropriate threshold values by choosing the point on the ROC curve closest to 0 on the x axis and on the y axis to discriminate dementia-free from dementia status. Specificity (SP), Sensitivity (SE), number (%) of subjects who did not need full neuropsychological assessment, number of dementia-free cases incorrectly categorized as dementia cases, PLR, and NLR were calculated and reported to identify the optimal usage of memory clinic resources.

## 3. Results

Of the 716 participants included in the present study, 488 were diagnosed as dementia patients, and 228 were dementia-free cases (172 MCI and 56 No Cognitive Impairment NCI).

As a result of the study setting (memory clinic), a high (68.2%) proportion of dementia patients was observed. Dementia patients were significantly older and less educated than dementia-free patients. There was no significant gender or ethnic differences (Table 1).

Table 2 and Table 3 present a range of MMSE cut-off scores with their respective PLR/NLRs before and after education stratification. We only report MMSE cut-off scores with either PLRs ranging from >5 till the highest), or NLRs ranging from <0.2 till the lowest).

**Table 1:** Demographics and Mini-Mental State Examination (MMSE) total scores in dementia and dementia-free groups.

Characteristics	Dementia-Free (*n* = 228)	Dementia (*n* = 488)	*p*
Age, mean (SD)	67.6 (11.1)	75.9 (8.3)	<0.001
Education, no or primary level, *n* (%)	48.7% (111)	72.8% (355)	<0.001
Gender, male, *n* (%)	44.3% (101)	45.3% (221)	0.81
Ethnicity, Chinese, *n* (%)	82.5% (188)	81.6% (398)	0.84
Non-Chinese, *n* (%)	17.5% (40)	18.4% (90)	
MMSE total score, mean (SD)	23.2 (5.2)	14.5 (5.3)	<0.001

**Table 2:** Area Under Curves (AUCs), MMSE cut-off scores, Specificity (SP), Sensitivity (SE), number (%) of patients correctly identified, number (%) of patients misclassified as dementia, and PLRs before and after education stratification.

Group	AUC (95% CI)	Cut-off Score	SP	SE	No. (%) of Patients Correctly Identified as Having Dementia	No. (%) of Dementia-Free Patients Misclassified as Dementia	PLR
Whole group (*n* = 716)	0.87 (0.84–0.90)	4/5 *	100%	2.9%	14 (2.0%)	0	-
		5/6	99.6%	4.5%	22 (3.1%)	1 (0.1%)	11.3
		6/7	99.1%	6.6%	32 (4.5%)	2 (0.3%)	7.3
		7/8	99.1%	8.2%	40 (5.6%)	2 (0.3%)	9.1
		8/9 ^Ϯ^	99.1%	11.9%	58 (8.1%)	2 (0.3%)	13.2
No or primary level of education (*n* = 474)	0.84 (0.80–0.89)	4/5 *	100%	3.0%	11 (2.3%)	0	-
		5/6	99.1%	5.2%	18 (3.8%)	1 (0.2%)	5.8
		6/7	98.2%	7.7%	27 (5.7%)	2 (0.4%)	4.3
		7/8	98.2%	9.9%	34 (7.2%)	2 (0.4%)	5.5
		8/9 ^Ϯ^	98.2%	14.6%	51 (10.8%)	2 (0.4%)	8.1
Secondary and higher level of education (*n* = 242)	0.89 (0.77–1.00)	9/10 *	100%	7.1%	9 (3.7%)	0	-
		10/11	99.1%	8.7%	11 (4.5%)	1 (0.4%)	9.7
		11/12	99.1%	13.5%	17 (7.0%)	1 (0.4%)	15.0
		12/13	99.1%	16.7%	21 (8.7%)	2 (0.8%)	18.6
		13/14	99.1%	20.6%	26 (10.7%)	2 (0.8%)	22.9
		14/15	98.3%	30.2%	35 (14.5%)	2 (0.8%)	17.8
		15/16	98.3%	35.7%	44 (18.2%)	2 (0.8%)	21.0
		16/17 ^Ϯ^	98.3%	40.5%	50 (20.7%)	2 (0.8%)	23.8

AUC, Area Under the Curve; SP, Specificity; SE, Sensitivity; PLR, Positive Likelihood Ratio; *, MMSE cut-off scores with no risk to misclassify dementia-free cases as dementia; ^Ϯ^, MMSE cut-off scores with the highest PLR.

**Table 3:** AUCs, cut-off scores of the MMSE, SP, SE, number (%) of patients correctly identified as dementia-free, number (%) of dementia subjects misclassified as dementia-free and NLRs before and after education stratification.

Group	AUC (95% CI)	Cut-off Score	SP	SE	No. (%) of Patients Correctly Identified as Dementia-Free	No. (%) of Dementia Subjects Misclassified as Dementia-Free	NLR
Whole group (*n* = 716)	0.87 (0.84–0.90)	21/22	68.4%	91.4%	156 (21.8%)	42 (5.9%)	0.1
		22/23	64.0%	94.1%	144 (20.1%)	28 (3.9%)	0.09
		23/24	57.9%	95.5%	131 (18.3%)	21 (2.9%)	0.08
		24/25	52.6%	97.1%	121 (16.9%)	16 (2.2%)	0.06
		25/26	45.6%	97.7%	104 (14.5%)	11 (1.5%)	0.05
		26/27	34.2%	98.6%	76 (10.6%)	7 (1.0%)	0.04
		27/28	22.4%	99.2%	50 (7.0%)	4 (0.6%)	0.04
		28/29 *	6.2%	100%	14 (2.0%)	0	0
No or primary level of education (*n* = 474)	0.84 (0.80–0.89)	21/22	46.4%	95.9%	51 (10.8%)	14 (3.0%)	0.09
		22/23	39.3%	98.3%	43 (9.1%)	6 (1.3%)	0.04
		23/24	30.4%	99.2%	35 (7.4%)	3 (0.6%)	0.03
		24/25	27.7%	99.4%	32 (6.8%)	2 (0.4%)	0.02
		25/26	22.3%	99.4%	25 (5.3%)	2 (0.4%)	0.03
		26/27	14.3%	99.7%	15 (3.2%)	1 (0.2%)	0.02
		27/28 *	6.3%	100%	7 (1.5%)	0	0
Secondary and higher level of education (*n* = 242)	0.89 (0.77–1.00)	24/25	76.7%	90.5%	89 (36.8%)	14 (5.8%)	0.1
		25/26	68.1%	92.9%	79 (32.6%)	9 (3.7%)	0.1
		26/27	53.4%	95.2%	55 (22.7%)	6 (2.5%)	0.09
		27/28	37.9%	96.8%	43 (17.8%)	4 (1.7%)	0.08
		28/29 *	21.6%	100%	25 (10.3%)	0	0

NLR, Negative Likelihood Ratio; *, MMSE cut-off scores with the lowest NLR and no risk to misclassify dementia cases as dementia-free.

**Figure 1:**
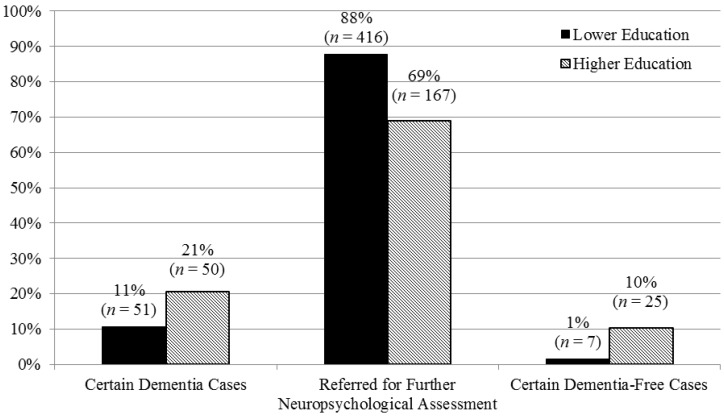
No. (%) of certain dementia cases, cases requiring further neuropsychological assessment, and certain dementia-free cases in lower and higher education groups.

Figure 1 shows numbers and percentages of certain dementia cases, cases requiring further neuropsychological investigation and certain dementia-free cases in education stratification groups using the optimal MMSE cut-off scores.

In the present study, a MMSE cut-off of 8/9 resulted in the highest PLR (13.2) for dementia among all patients. Using this cut-off score, a total of 8.1% of the whole sample were correctly classified as dementia patients. In contrast, only 0.3% of the entire sample was misdiagnosed as dementia cases when not having dementia (Table 2).

ANOVA showed a significant effect of educational level (F_(1, 706)_ = 40.46, *p* < 0.001) on MMSE performance, independent of clinical consensus diagnosis (Education × Diagnosis interaction: F_(1, 706)_ = 2.75, *p* = 0.06). Hence optimal PLRs/NLRs for MMSE cut-off scores were investigated at different educational levels (no or primary level of education, or secondary and higher level of education). After educational stratification, a cut-off of 16/17 rendered the optimal PLR of 23.8 amongst patients educated beyond primary level with a total of 20.7% (50 out of 242) correctly identified as dementia cases, whereas 0.8% (2 out of 242) were wrongly classified (Figure 1).

In patients with primary or less education, the optimal PLR (8.2) was achieved with a MMSE cut-off of 8/9 which resulted in 10.8% (51 out of 474) been correctly identified as dementia cases, whereas 0.4% (2 out of 474) were wrongly classified (Figure 1).

In contrast, a MMSE cut-off score of 28/29 provided the optimal NLR (0), correctly identifying 2% (14 out of 716) of the whole sample as dementia-free patients (Table 2). After education stratification, a MMSE cut-off of 27/28 achieved the optimal NLR among patients with no or primary level of education, with 1.5% (7 out of 474) correctly classified as dementia-free cases. In patients with secondary and above level of education, the optimal NLR was at a MMSE cut-off of 28/29, resulting in 10.3% (25 out of 242) correct identification rate (Table 3).

Overall, before education stratification, applying two MMSE cut-off points with the optimal PLR (8/9) and NLR (28/29), 10.1% (72 out of 716) of the whole sample would not need to undergo further neuropsychological assessment, accompanied by a 0.3% (2 out of 716) incorrect classification rate (dementia-free patients categorized as dementia patients). After education stratification, 18.6% (133 out of 716) of the whole sample would not require neuropsychological assessment, with a 0.6% (4 out of 716) incorrect categorization rate (Table 4).

**Table 4:** Total number (%) of patients who do not need to undergo full neuropsychological assessment, number (%) of dementia-free patients categorized as dementia patients.

Group	Education Stratification	Cut-off Scores	Optimal PLR		Cut-off Scores	Optimal NLR	
			No. (%) of patients not requiring neuropsychological assessment	No. (%) of dementia-free patients incorrectly categorized as dementia patients		No. (%) of dementia-free subjects not requiring neuropsychological assessment	No. (%) of dementia patients incorrectly categorized as dementia-free
Before stratification	-	8/9	58 (8.1%)	2 (0.3%)	28/29	14 (2.0%)	0
	-						
After stratification	no or primary level	8/9	101 (14.1%)	4 (0.6%)	27/28	32 (4.5%)	0
	Secondary and above level	16/17			28/29	-	

## 4. Discussion

The principal finding of the present study is that education stratified MMSE cut-off scores can be used to correctly exclude dementia and dementia-free patients from requiring further neuropsychological evaluation with minimal misclassification rate.

The MMSE has been widely used in memory clinic settings for screening and long term care purpose, even though it is now copyrighted. However other brief memory tests, such as the Montreal Cognitive Assessment (MoCA) and the Hopkins Verbal Learning Test (HVLT), are also widely used to provide accurate screening for MCI and dementia [26,27,28]. Further studies are required to compare the discriminating ability of the MMSE with other brief cognitive instruments. In addition, it is worth mentioning that the MMSE should not be used solely to replace comprehensive neuropsychological assessment for diagnostic purposes. Patients who attend memory clinic are often those who express memory complaint and sometimes referred from primary healthcare centers, and a comprehensive neuropsychological assessment would provide more insight on deficits in both global and domain-specific cognitive function. The additional information is useful in aiding clinician in clinical evaluation and case management [29]. Furthermore, standard clinical examination and clinician’s clinical judgment is crucial in making dementia diagnosis. In addition, there is a notable percentage of MCIs in our study (24%). Although we did not specify MMSE cut-off scores for MCI group as it is not the targeted population in the current study, we do recognize that the MMSE has limited value in detecting MCI. Therefore we suggest that the MCI patients should go through an extensive neuropsychological assessment.

The present study is the first to explore educational- specific MMSE cut-off scores for exclusion purpose in the memory clinic setting. Previously, a memory-clinic based study in Germany investigated the psychometric properties of the MMSE together with other dementia screening tests and reported a MMSE cut-off score with 100% specificity of 23/24 in detecting dementia successfully identified 79.9% of the dementia patients [30]. However the sample size of that study is small (*n* = 123) with a younger mean age of 69 (ranging from 44 to 90); the education level of participants was not included in the report. Another German study also used the MMSE as a dementia screening tool and reported a cut-off score of ≤25 with 100% specificity in detecting dementia patients (86.8% of dementia cases been correctly categorized) [31]. However, our results are not comparable to these 2 Germany studies as we applied a unique approach in deriving MMSE cut-off scores to be used to exclude dementia and dementia-free cases using optimal PLR and NLR value to select MMSE cut-off scores. Furthermore, neither previous study took education level into account when investigating the discriminant ability of the MMSE, even though MMSE performance is known to be affected by education [32,33,34]. This may due to the overall high level of education among participants (10.7 years of education for dementia patients compared to.12.2 years of education for controls) [31]. When the MMSE was applied to investigate its dementia discriminant ability among highly educated individuals (16 years and more), an optimal cut-off score of 27 was obtained with a PLR of 9.6, resulting in 67% of the dementia patients been correctly identified [35]. As educational disparities are common in the developing world [36], it is essential that studies conducted in these countries explore education specific MMSE cut-off scores. The use of such cut-offs enabled the identification of up to 18.6% (133 of 716) patients in this study who did not require further neuropsychological evaluation—with a low misclassification rate of 0.56% (4 out of 716).

Two earlier community-based studies have reported on MMSE educational cut-off scores in detecting MCI/dementia in Singapore [14,18]. One study reported that with a cut-off score of 23/24, higher specificity (85.2%) were found in higher educated subjects (Secondary and higher education), as compared with subjects with lower level of education (specificity 57.3%; education: none and primary) in discriminating between dementia and dementia-free patients [14]. Another community-based study found that to detect early cognitive impairment, optimal MMSE cut-off scores of 25, 27 and 29 were obtained for different education groups (none, primary and secondary and above) [18]. Nevertheless results from these 2 studies were not comparable to the present study due to differences in study setting and optimal MMSE cut-off deriving approach.

Although both previous Singapore based studies suggest ethnicity, education level and age should be taken into account in the interpretation of optimal MMSE cut-offs, we did not find ethnic differences on MMSE performance. Furthermore, we found that optimal MMSE cut-off points we derived after age and education stratification were at no difference from what we obtained after education stratification. Therefore we omitted age stratification from our current analyses.

## 5. Limitation

There are several limitations of the present study. Firstly, this is a single center, clinic based study. Thus, the generalizability of the current study to epidemiological studies may be limited, as the proportion of dementia patients in the community setting is lower [14,18,37]. Secondly, we did not examine ethnic differences on MMSE performance due to the small size of the non-Chinese group (18.2%). It is suggested that ethnic non-equivalence should be taken into consideration when administering the MMSE among less educated (no or primary level of education) participants. Although we did not find ethnic difference on MMSE performance in the current study, future studies should further examine ethnic differences on MMSE performance.

## 6. Conclusions

In conclusion, the MMSE has utility in excluding definite dementia and dementia-free cases from requiring further neuropsychological examination in the memory clinic setting, in addition to its usual utility in screening for possible dementia cases [1]. It provides clinicians with a means of using a brief cognitive test to exclude the need for more extensive neuropsychological assessment and to expedite the process of clinical evaluation for the diagnosis of dementia. This new means of using the MMSE would allow patients to receive hospital service at a better speed with a lower cost.
